# A hierarchical nest survival model integrating incomplete temporally varying covariates

**DOI:** 10.1002/ece3.822

**Published:** 2013-10-10

**Authors:** Sarah J Converse, J Andrew Royle, Peter H Adler, Richard P Urbanek, Jeb A Barzen

**Affiliations:** 1 USGS Patuxent Wildlife Research Center12100 Beech Forest Road, Laurel, Maryland; 2 Entomology Program, Clemson UniversityClemson, South Carolina; 3 USFWS Necedah National Wildlife RefugeGrand Dike Road, Necedah, Wisconsin; 4 International Crane FoundationE11376, Shady Lane Road, Baraboo, Wisconsin

**Keywords:** Autoregressive model, Bayesian analysis, black fly, Culicidae, daily nest survival, dynamic occupancy, *Grus americana*, reintroduction, Simuliidae, Tabanidae, whooping crane

## Abstract

Nest success is a critical determinant of the dynamics of avian populations, and nest survival modeling has played a key role in advancing avian ecology and management. Beginning with the development of daily nest survival models, and proceeding through subsequent extensions, the capacity for modeling the effects of hypothesized factors on nest survival has expanded greatly. We extend nest survival models further by introducing an approach to deal with incompletely observed, temporally varying covariates using a hierarchical model. Hierarchical modeling offers a way to separate process and observational components of demographic models to obtain estimates of the parameters of primary interest, and to evaluate structural effects of ecological and management interest. We built a hierarchical model for daily nest survival to analyze nest data from reintroduced whooping cranes (*Grus americana*) in the Eastern Migratory Population. This reintroduction effort has been beset by poor reproduction, apparently due primarily to nest abandonment by breeding birds. We used the model to assess support for the hypothesis that nest abandonment is caused by harassment from biting insects. We obtained indices of blood-feeding insect populations based on the spatially interpolated counts of insects captured in carbon dioxide traps. However, insect trapping was not conducted daily, and so we had incomplete information on a temporally variable covariate of interest. We therefore supplemented our nest survival model with a parallel model for estimating the values of the missing insect covariates. We used Bayesian model selection to identify the best predictors of daily nest survival. Our results suggest that the black fly *Simulium annulus* may be negatively affecting nest survival of reintroduced whooping cranes, with decreasing nest survival as abundance of *S. annulus* increases. The modeling framework we have developed will be applied in the future to a larger data set to evaluate the biting-insect hypothesis and other hypotheses for nesting failure in this reintroduced population; resulting inferences will support ongoing efforts to manage this population via an adaptive management approach. Wider application of our approach offers promise for modeling the effects of other temporally varying, but imperfectly observed covariates on nest survival, including the possibility of modeling temporally varying covariates collected from incubating adults.

## Introduction

Nest success – the probability that a nest will produce at least one individual – is a key vital rate affecting the evolution, ecology, and management of avian populations. Nest success has long been a focus of study for avian ecologists, and associated statistical methods have been in development for more than 50 years. Mayfield's (1961, 1975) work was a major breakthrough, developed to address the problem that apparent nest success (the proportion of sampled nests that are successful) will be a biased measure of true nest success for most sampling scenarios. Nests that are lost early in incubation tend to be underrepresented in samples, as nests are less likely to be detected after they fail. To address this, the Mayfield method instead considers daily nest survival (*S*):


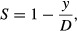


where *y* is the number of nest failures observed, and *D* is exposure days – the sum across nests of days in which each nest is monitored, from initial detection to termination. However, Mayfield's method requires that the date of nest failure be known, which will not be achieved when the interval between nest checks is >1 day. Alternatively, Mayfield assumed that unsuccessful nests failed halfway through the terminal observation interval, thus allocating to exposure days half the number of days in the interval. Johnson (1979), Hensler and Nichols (1981), and Bart and Robson (1982) developed likelihood functions for daily nest survival [reviewed by Williams et al. (2002)] that addressed the problem of uncertain failure date. Subsequently, Dinsmore et al. (2002), Stephens (2003), and Shaffer (2004) developed generalized linear models allowing for flexible modeling of variation in daily nest survival [reviewed by Rotella et al. (2004)]. Bayesian developments have also been presented (Royle and Dorazio 2008; Schmidt et al. 2010). In the above cases, the mortality hazard rate is assumed to be constant over the life of the nest (i.e., age-constant survival), or if age-dependent variation in survival is of interest, the assumption is that nests can be aged without error at first detection (e.g., Dinsmore et al. 2002). The case of age- or stage-dependent survival with unknown nest age has also been considered (Heisey and Nordheim 1995; He et al. 2001; Pollock and Cornelius 2001; He 2003; Stanley 2004; Cao et al. 2008).

We focus here on the case of age-constant nest survival and extend nest survival models to handle incompletely observed temporally varying covariates. A challenge with covariates of this type arises frequently in survival analysis under mark–recapture designs, when temporally varying covariates associated with an individual (e.g., body mass, reproductive condition) cannot be observed when the individual is not captured. Modeling the impact of such covariates on survival, then, has long been a technical challenge (Pollock 2002), and a handful of solutions have emerged (Nichols et al. 1992; Bonner and Schwarz 2006; Catchpole et al. 2008; Langrock and King 2013). We developed an extension of nest survival models to handle incompletely observed temporally varying covariates. Our extension was motivated by a case study involving nest survival in whooping cranes (*Grus americana*).

The reintroduction of the Eastern Migratory Population of whooping cranes to central Wisconsin (largely on Necedah National Wildlife Refuge; NNWR) is a cornerstone effort in whooping crane conservation. Adding an additional 1 or 2 whooping crane populations is a goal of the Whooping Crane International Recovery Plan (Canadian Wildlife Service & U.S. Fish and Wildlife Service 2005). Many indicators of success for this reintroduced population, established with releases of captive-reared birds beginning in 2001, are good (Urbanek et al. 2009; Converse et al. 2012). However, reproductive success has been poor (only 20 of 109 nests, through 2012, produced a hatchling), largely due to a high rate of nest abandonment.

In 2008, RP Urbanek posed the hypothesis that nest abandonment is caused by harassment of cranes by blood-feeding black flies of the genus *Simulium* (Urbanek et al. 2010). Since that time, regular collection of insect index data has been conducted on NNWR using carbon dioxide traps. However, insect sampling is logistically challenging (e.g., transporting dry ice to remote trap locations) and time-consuming (e.g., sample processing). Therefore, sampling is conducted less than once per day. While the intensive sampling of this small, reintroduced, whooping crane population largely obviates the original motivation for development of the Mayfield method – essentially all nests are located within 1–2 days of initiation – the focus of estimation is on daily nest survival because the temporal pattern of nest failure may be key to understanding the cause of nest failure. To carry out the analysis for the biting-insect hypothesis, we developed a novel daily nest survival model to account for missing insect population indices from carbon dioxide traps. The goal of this article is to describe and demonstrate the model with a subset of the whooping crane nest survival data (2009–2010). We also demonstrate the use of Bayesian model selection, which allows us to distinguish among a relatively large set of potentially predictive insect population metrics. The method described herein will be implemented for the full nest survival data set for this population upon the completion of ongoing monitoring and experimentation.

## Methods

### Study population and location

The Eastern Migratory Population of whooping cranes was established via releases of captive-reared birds in 2001 and every year since. The majority of birds in the population were reared and trained to migrate via ultralight aircraft-led migrations in their first fall (Urbanek et al. 2005) between NNWR in central Wisconsin and the Gulf Coast of Florida (Chassahowitzka National Wildlife Refuge and St Marks National Wildlife Refuge). Beginning in 2005, an additional release type, direct autumn release, was initiated, wherein birds were released in Wisconsin during their first fall in the vicinity of older birds, with the intention that older birds would teach younger birds the migratory pathway. On 1 April 2009, there were 73 birds in the EMP, and by 1 April 2010, there were 89 birds.

Whooping cranes nest in wetland habitats and construct large (>1 m diameter) nests from emergent vegetation (Allen 1952; Kuyt 1995). Whooping crane nests most frequently contain 2 eggs, although occasionally a nest will contain only 1 egg, and rarely 3 eggs (Kuyt 1995). Incubation responsibilities are shared by the male and female (Kuyt 1995). The incubation period is 28–34 days (Gabel and Mahan 1996), most commonly 29–30 days (Kuyt 1982). We assume a 30-day incubation period.

All nests described herein were on or in the vicinity of NNWR, a 177-km^2^ US Fish and Wildlife Service-owned National Wildlife Refuge in central Wisconsin, USA, northwest of the town of Necedah. NNWR is composed of a combination of large wetland complexes intermixed with upland grassland and woodland, with minimal topographic relief.

### Nest survival monitoring

We used data from all known whooping crane nests (*n* = 34) in the Eastern Migratory Population, 2009–2010. For successful nests, we terminated the encounter history as soon as an egg hatched, such that nests that produced any live hatchling were considered successful (whooping cranes typically lay 2 eggs, which hatch asynchronously; Kuyt 1995). Nest initiation was determined via direct observation (ground-based or aerial) and/or radiotracking of birds. The intensity of monitoring was sufficient to make it unlikely that any nests went undetected. Monitoring after detection consisted of a combination of ground-based or aerial observation and radiotracking of adult birds (to determine whether they were still attending the nest). Nest checks were most often daily. In some cases, intervals between checks were longer, but only rarely longer than 3 days. Also, in some cases, nest fate was determined after the fact based on video cameras deployed just within range of nests.

### Insect monitoring

To build the nest survival model, we used data from 7 carbon dioxide traps located around NNWR in 2009. All 7 traps were operated in 2009, but in 2010, only three of the traps were operated. It was possible to fill in zeros for missing insect data from before and after trapping commenced each year in some cases, if it was known for certain that adults of a particular insect species had not yet emerged (based on the monitoring of the insect breeding sites).

Insect data sets were compiled in three different ways for use in the nest survival models, resulting in three predictor variables for each of the insect taxa. To construct the variables, we first spatially interpolated counts for each taxon, specific to each nest *i* on day *t*, using the inverse-distance-squared interpolator:


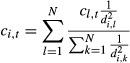


where *c*_*l,t*_ is the count at trap *l* on day *t*, *d*_*i,l*_ is the distance between nest *i* and trap *l*, and *N* is the total number of traps active on day *t*. If, on a particular date, data were available from some traps, while other traps were inactive (e.g., due to the trap being blown over by wind gusts), weighted means were calculated from the remaining traps.

Once we had nest-specific counts, we constructed 3 different metrics to describe insect populations. The metrics were developed to reflect alternative hypothesized relationships between nest survival and insect populations. First, we used the counts themselves, transformed as *ln*(count + 1). Second, we used a presence/absence indicator (equal to 1 on any day where the interpolated count was >0, that is, any day in which insects were detected in any trap). Finally, we used an indicator for any day when the nest-specific weighted count exceeded the 90% quantile for the entire count data set (for all nests), that is, the days when interpolated counts at a nest were particularly high.

We considered four different insect taxa in the analysis. First, we included *S. annulus* and *S. johannseni*, the two most abundant ornithophilic black flies in the carbon dioxide samples. We also included two additional taxa of blood-feeding insects that were common and widespread in the insect survey data, including mosquitoes (family Culicidae) and horse flies (family Tabanidae).

### Nest survival model

There are two basic components to the model that we developed. First is the nest survival portion of the model. Data for this portion of the model consist of the nest encounter history *X*_*i,t*_ for nest *i* on day *t*, where *X*_*i,t*_ = 1 if the nest was observed alive, 0 if the nest was observed dead, and “NA” if the nest was not observed (i.e., coded as missing data). We assumed (reasonably so) that successful nests were observed on the day of hatching, so the nest record would terminate once the first egg hatched. In 2010, two nests did not hatch, although they were incubated full term (eggs were either infertile or the embryos died). We assumed that these nests failed on the 30th day of incubation – this is the typical incubation period for whooping cranes (Kuyt 1995). Other assumptions could be made, but because nest abandonment appears to be the major proximal cause of nest failure, we decided to treat the nest as successful until the end of incubation, as it was not abandoned before that point. Starting with the first day after nest detection, the nest encounter history *X*_*i,t+1*_ is distributed as follows:


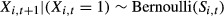


and


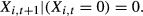


Then,


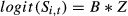


where *S*_*i,t*_ is the survival probability for nest *i* on day *t*, the *B* are model parameters, and the *Z* are a set of predictor variables, which may include *I*_*i,t+1*_ – the day- and nest-specific insect predictors. We included a random intercept for each nesting pair, so β_0_[pair_*i*_]∼ Normal (μ_pair_, σ_pair_), and we also included a fixed effect of renesting, applied as an indicator to second and third nests, in addition to the insect variables included as described below.

The second portion of the model considers the insect populations. For count data – transformed as *ln*(count +1) – we modeled:


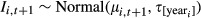


where τ_*y*_ is a year-specific precision term for *y* = 1:2 (2009 and 2010), and


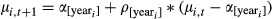


where *α*_*y*_ is a year-specific mean, and *ρ*_*y*_ is a year-specific autoregressive parameter. Alternatively, for the 2 types of indicator data (presence/absence and >90% quantile), we used a model analogous to the process portion of a dynamic occupancy model (MacKenzie et al. 2003), such that


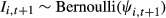


and


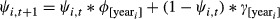


In this case, *φ* is a year-specific patch survival term and γ is a year-specific patch colonization term, where, in this case, a patch is a nest. In other words, a nest occupied by insects (i.e., where insects were predicted to be present) on the previous day is subject to a survival probability, and a nest unoccupied on the previous day is subject to a colonization probability.

### Bayesian model selection

To facilitate inference about the predictive value of the different insect indices for daily nest survival, we conducted Bayesian model selection using the Kuo and Mallick (1998) indicator variable approach (see also Link and Barker 2006; Royle and Dorazio 2008; Smith et al. 2011). We considered, with equal prior weight, all possible models given the 12 insect variables, producing 2^12^ possible models. To achieve this, we modeled each of the insect variables in parallel in the analysis. We then included the full set of insect effects in the linear predictor for daily nest survival. However, we also included, associated with each insect effect, an indicator variable, *w*_*m*_ for the insect variables 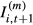 such that:


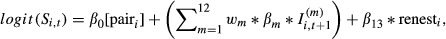


where the prior for *w*_*m*_∼Bernoulli (0.5). Each sample from the Markov chain Monte Carlo (MCMC) algorithm then included an indicator for whether a given variable was included in the model: 1 if the variable was included in the model and 0 if it was not. We then calculated the Bayes factor (BF) for each insect variable from the prior mean of *w*_*m*_ (0.5) and the posterior mean of *w*_*m*_ (*w*_*m*_|data) as follows:


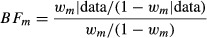


In other words, the BF is the odds ratio for inclusion of the variable in the model (Smith et al. 2011). Because the prior distributions on model parameters can influence Bayesian model selection results, Link and Barker (2006) recommend that the total prior uncertainty should remain constant regardless of the dimensions of the model. We achieved this by scaling the variance of the parameter prior distributions. We gave each of the β_*m*_ coefficients a mean zero normal prior, with a variance equal to *V*/*K*, where *K* was equal to the number of insect effects entering the model at a particular sample (i.e., 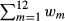). We then placed a Gamma-distributed prior on the total variance of the linear predictor, *V*, with parameters 3.29 and 7.8 (Link and Barker 2006). This prior results in a marginal distribution for nest survival that is approximately Uniform(0,1).

### Model fitting

We fit the models using MCMC methods implemented in JAGS (Plummer 2012; use of trade or product names does not imply endorsement by the US government) via R (R Development Core Team 2004) and the R package rjags (Plummer 2013; an R script for running the model can be obtained from the corresponding author). We used standard flat priors for all terms in the model (except as described above), including Gamma (0.1,0.1) for the inverse of variance terms, Uniform(0,1) priors for the parameters in the dynamic occupancy-type insect models, and normal priors with mean = 0 and variance = 1000 otherwise. We sampled from three independent Markov chains a total of 500,000 samples after discarding the first 20,000 samples. We chose such a large number of samples because of the large number of models implied by the model selection procedure, 2^12^. This allowed us to obtain enough samples under a single model for reasonable model-conditional posterior distributions of effects of interest. We evaluated the behavior of the MCMC routine based on the visual inspection of chains and on 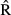 < 1.05, as recommended by Gelman et al. (2004).

## Results

**Figure 1:**
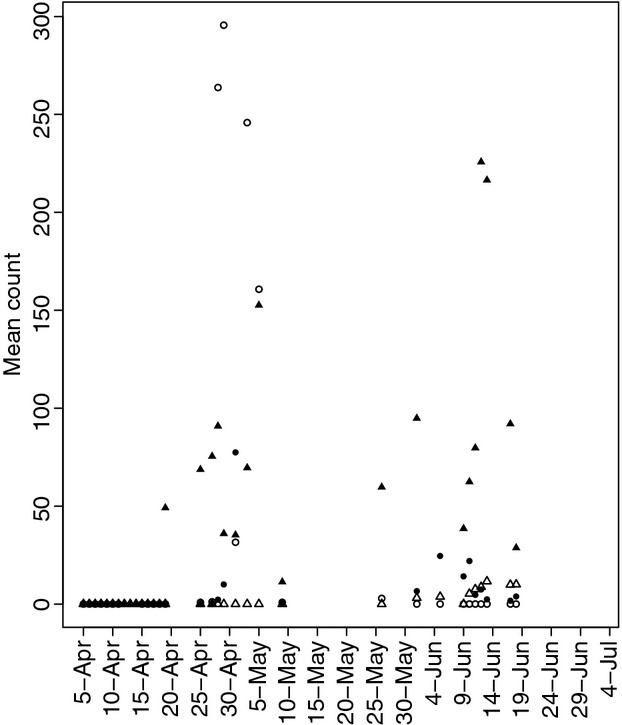
Mean counts of four insect taxa from 7 carbon dioxide traps deployed on Necedah National Wildlife Refuge in spring 2009. Taxa include *Simulium annulus* (open circles), *S. johannseni* (closed circles), horse flies (open triangles), and mosquitoes (closed triangles).

**Figure 2:**
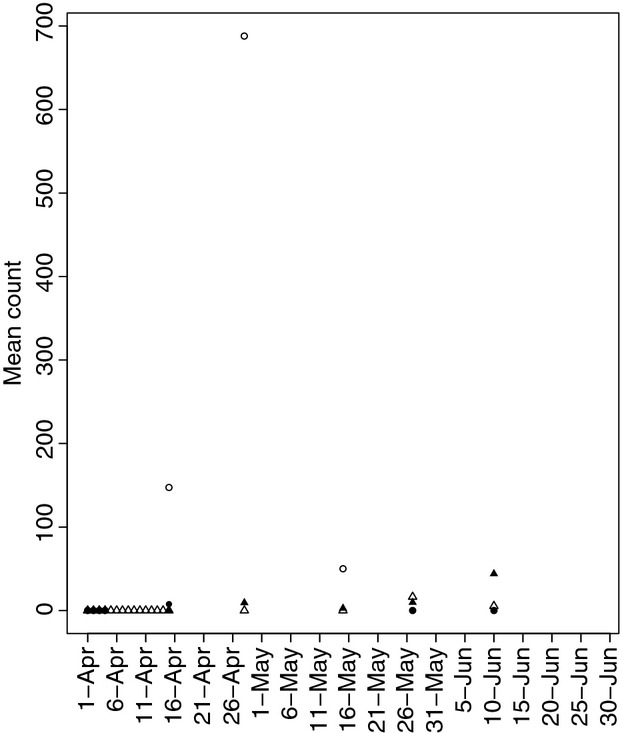
Mean counts of four insect taxa from three carbon dioxide traps deployed on Necedah National Wildlife Refuge in spring 2010. Taxa include *Simulium annulus* (open circles), *S. johannseni* (closed circles), horse flies (open triangles), and mosquitoes (closed triangles).

In 2009, insect trapping was conducted on 50 days between 4 April and 15 June, the period when nests were active. In 2010, trapping was less frequent than in 2009 (2010: 5 days of trapping between 1 April and 14 June). In both 2009 and 2010, the highest counts of the four taxa were for *S. annulus*, and these high counts occurred near the end of April (Figs 1 and 2). In 2009, relatively high counts of mosquitoes were also observed, in mid-June. The relative infrequency of sampling in 2010 made it more difficult to see clear increases and decreases in the insect counts.

Of the 34 nests included here (*n* = 17 in each year), only 7 produced chicks (2 in 2009, 5 in 2010). There were 5 second nests attempted in 2009 (i.e., 12 pairs nested in 2009), and in 2009, there were 4 second nest attempts and 1 third nest attempt (12 pairs nested in 2010). In addition to the 7 successful nests, an additional 2 nests (both in 2010) were incubated >30 days, but failed due to infertility or because embryos died during incubation. Of the 7 nests that produced chicks, only two of them were first nesting attempts, both in 2010. These 2 nests were initiated later in the nesting season than any other first nest observed.

**Figure 3:**
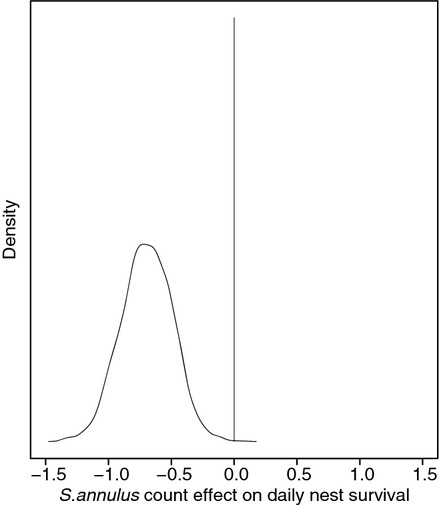
The posterior distribution of the effect, on the logit scale, of *ln*-transformed counts of the black fly *Simulium annulus*, based on the spatially interpolated counts from carbon dioxide traps, on daily nest survival in the Eastern Migratory Population of whooping cranes, 2009–2010.

Of the 12 insect variables we considered as predictors of daily nest survival, only one had a BF > 3 (Table 1) – the *ln-*transformed counts of *S. annulus*. This variable had a posterior inclusion probability of 0.92, whereas all other variables had a posterior inclusion probability <0.75 and a BF < 3. The effect estimate for the *S. annulus* count variable was strongly negative (β = −0.695, 95% CI = −1.097, −0.309; Fig. 3). This estimate is conditional on the top-ranked model including the *S. annulus* count variable, that is, the model that was sampled the greatest number of times (based on the Bernoulli *w*_*m*_ variables) in the MCMC routine.

**Table 1:** Model selection results for 12 insect variables hypothesized to affect daily nest survival in the Eastern Migratory Population of whooping cranes. The posterior inclusion probability is the probability that the variable should be included in the model, and the Bayes factor (BF) is the posterior odds ratio in favor of the set of models including the variable versus the set of models not including the variable

Insect Variable^1^	Posterior inclusion probability^2^	Bayes Factor^3^
*S. annulus* ln(count+1)	0.92	10.80
*S. annulus* presence	0.52	1.09
*S. annulus* >90% quantile	0.50	0.99
*S. johannseni* ln(count+1)	0.74	2.84
*S. johannseni* presence	0.50	0.99
*S. johannseni* >90% quantile	0.50	0.99
Tabanidae ln(count+1)	0.46	0.85
Tabanidae presence	0.59	1.41
Tabanidae >90% quantile	0.52	1.08
Mosquitoes ln(count+1)	0.24	0.32
Mosquitoes presence	0.51	1.02
Mosquitoes >90% quantile	0.48	0.91

1 Insect variables included, for each of 4 taxa, *ln*-transformed counts at a nest, an indicator for presence, and an indicator for days when the count exceeded the 90% quantile of all counts.

2 Posterior mean of the *w* variables described in the text.

3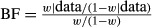, where w|data is the posterior inclusion probability, and *w* is the prior inclusion probability = 0.5.

**Figure 4:**
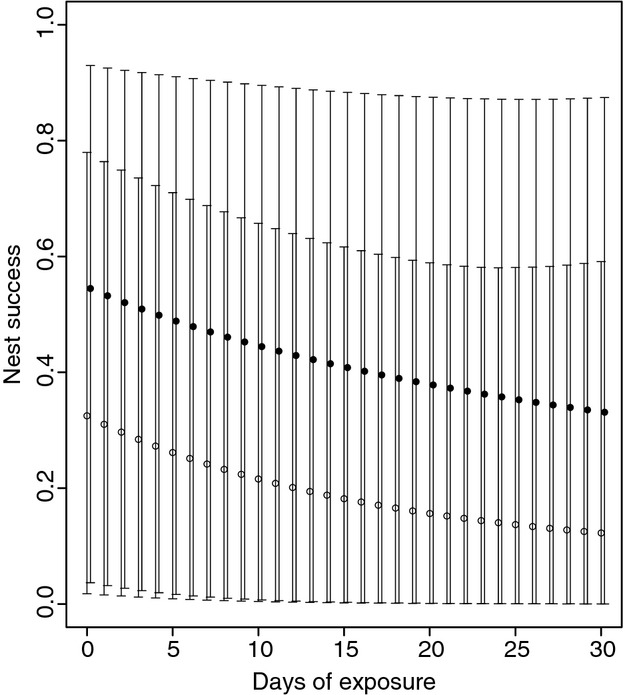
Predicted probability of nest success (the probability that a nest produces ≥1 hatchling) based on the days of exposure to the mean level of counts of the black fly *Simulium annulus* in the Eastern Migratory Population of whooping cranes, 2009–2010. Days of exposure = 0 is the probability of success with no black fly exposure during a 30-day incubation period, while days of exposure = 30 is the probability of success if a nest was exposed to black flies throughout its incubation period. Open circles are predicted probabilities for first nesting attempts, and Closed circles are predicted probabilities of success for renesting attempts. Error bars reflect 95% credible intervals.

Predicted daily probability of nest survival was 0.95 (95% CI = 0.87–0.99) for first nests not exposed to black flies and 0.90 (0.75–0.98) for first nests exposed to *S. annulus* at the mean observed level. For renesting, the equivalent probabilities were 0.97 (0.90–1.00) and 0.94 (0.75–1.00). Predicted probability of producing at least 1 hatchling (survival throughout a 30-day incubation period) for first nests varied between approximately 0.32 for nests never exposed to black flies to 0.12 for nests exposed to black flies at the mean observed level throughout incubation (Fig. 4). The corresponding values for renesting attempts were 0.54 and 0.33 (Fig. 4).

## Discussion

Flexible, generalized linear modeling approaches to estimation of daily nest survival have contributed substantially to the study of nesting ecology in recent years (e.g., Dinsmore et al. 2002; Jehle et al. 2004; Rotella, Dinsmore & Shaffer 2004, 2004; Grant et al. 2005; Hood and Dinsmore 2007; Schmidt et al. 2010). These methods require fewer assumptions than the original formulation of Mayfield (1961, 1975) and are able to accommodate inclusion of temporal covariates (e.g., weather effects), fixed individual covariates (e.g., habitat), and individual covariates that change in a deterministic way (e.g., nest age).

In our case study, the daily nest survival modeling framework needed to be extended to accommodate temporally varying individual covariates, where the values of covariates were periodically unavailable. These covariates were specific to individual nests because insect counts were spatially interpolated. The high spatial variability in insect densities rendered spatial modeling of insect populations infeasible, and so instead, we used the distance-averaged counts. However, we note that similar analyses using insect counts only from the trap nearest the nest (and in 2010, this included only three possible traps) resulted in qualitatively equivalent inference, suggesting that trap-based counts are reasonably robust predictors of daily nest survival.

Our problem is analogous to that addressed by Bonner and Schwarz (2006), who considered the problem of modeling survival using the Cormack–Jolly–Seber capture–recapture model (Cormack 1964; Jolly 1965; Seber 1965) with a temporally varying individual covariate (see Pollock 2002 for a general discussion of the issue). In that case, the missing data arise because the covariate cannot be observed when the individual is not captured. Here, our missing covariates arise due to the less-than-daily frequency of data collection on the covariate. The conceptual approach, however, is similar: a Markovian model is used to describe the covariate, and the missing data are sampled using MCMC methods. One can imagine myriad applications of this or similar models for temporally varying but imperfectly observed nest covariates, including covariates that relate to the condition of the nesting individual (e.g., a nesting bird's body mass or physical condition). Daily collection of such a covariate could itself have negative effects on nest success, so less frequent data collection may well be warranted. The key for completing such an analysis will be identifying an appropriate model for the missing data; we demonstrated two here, one for normally-distributed count data, and one for Bernoulli-distributed indicator data.

Bayesian model selection to identify the strongest predictor of daily nest survival, among the insect indicators considered, allowed us to more rigorously assess the evidence for particular insect population-based predictors of nest survival. Based on the posterior inclusion probabilities and Bayes factors (BFs), we had support for essentially only one insect variable, the *ln*-transformed counts of *S. annulus* populations. In our case, the BF represents evidence for a particular variable rather than a particular model, as the BF was calculated based on all models including a given variable versus all models excluding that variable (Smith et al. 2011). Jeffreys (1961) suggested that a BF between 3 and 12 indicated some support for a model, while a BF over 12 indicated strong support. This general guideline may prove useful in interpreting our results for readers unfamiliar with Bayesian model selection, although we caution against over-reliance on arbitrary cutoffs. As clearly articulated by Link and Barker (2010), strength of evidence represents a continuum. We interpret the BF of 10.8 to represent moderate to strong support in favor of the biting-insect hypothesis, when insects are represented as *ln-*transformed counts of *S. annulus*.

We found that even with relatively few nests and low insect sampling frequency (especially in 2010), we were able to demonstrate a link between *S. annulus* counts and daily nest survival in reintroduced whooping cranes. King et al. (2013) performed an analysis of daily nest survival for this population, but did not make use of the consistent and continuous data stream available from carbon dioxide traps. The provenance of the indicators of insect populations used in that analysis is unclear (to us), but apparently did not include nest-specific temporally varying predictors arising from a single, consistent, monitoring technique; however, we note that these authors also found a negative relationship between *S. annulus* abundance and daily nest survival. Whether the relationship we report here holds with a larger data set will be of primary interest in future investigations. In 2011 and 2012, a bacterial larvicide (*Bacillus thuringensis israelensis*; Bti) was applied to riverine habitat surrounding NNWR. Data from those years, as well as 2013 – a post-treatment year – will be analyzed to examine evidence for several different hypotheses for poor nest survival in this population.

Poor breeding success has rendered this reintroduced population nonviable (Converse et al. 2012; S. Servanty, Colorado State University, unpubl. data). Uncertainty in the appropriate management actions to take for this population is due to uncertainty in the cause of nest failure (Runge et al. 2011). Adaptive management is therefore the most appropriate process for decision-making in this reintroduced population (Williams et al. 2007; Runge 2011; McCarthy et al. 2012; Converse et al. 2013), and continued analysis of nest survival will be key in reducing uncertainty over time within an adaptive management process.
